# Comparison of Flavor Characteristics and Metabolite Basis of Oolong Tea from Six Different Tea Plant Cultivars Under High- and Low-Altitude Conditions

**DOI:** 10.3390/plants15010023

**Published:** 2025-12-21

**Authors:** Yuting Li, Shuaibo Shao, Siwei Deng, Zhendong Zhang, Yu Pan, Xingyuan Yao, Chengzhe Zhou, Zhong Wang, Yuqiong Guo

**Affiliations:** 1 Anxi College of Tea Science, Fujian Agriculture and Forestry University, Anxi County, Quanzhou 362400, China; liyuting2001@foxmail.com (Y.L.); bonight@163.com (S.S.); 19198027213@163.com (S.D.); xingyuanyao@fafu.edu.cn (X.Y.); chengzhechou@foxmail.com (C.Z.); zhongwang@fafu.edu.cn (Z.W.); 2 College of Horticulture, Fujian Agriculture and Forestry University, Fuzhou 350002, China; zzd0110@foxmail.com (Z.Z.); qq562523694@163.com (Y.P.); 3 Fujian Collaborative Innovation Center for Green Cultivation and Processing of Tea Tree in Universities, Fujian Agriculture and Forestry University, Anxi County, Quanzhou 362400, China

**Keywords:** *Camellia sinensis *(L.) O. Kuntze, GC-MS, macro-compositions, volatile organic compounds, quality

## Abstract

Oolong tea presents notable variations in taste profile and aroma characteristics under different cultivation conditions, particularly across altitudes. However, systematic investigations into the altitude-induced differences in key taste compounds and aroma composition remain limited. In this study, we examined six oolong tea cultivars, comparing their taste-related chemical constituents and aroma profiles under high- and low-altitude cultivation. Sensory evaluation, high-performance liquid chromatography (HPLC) and headspace solid-phase microextraction-gas chromatography-mass spectrometry (HS-SPME-GC-MS) were employed to characterize these differences. Sensory evaluation revealed that high-altitude oolong teas exhibited enhanced umami, sweetness, and floral intensity. In most cultivars, the levels of free amino acids, polyphenols, and soluble sugar were relatively higher under high-altitude conditions. HS-SPME-GC-MS identified 55 common volatile organic compounds (VOCs), with terpenes and esters comprising the largest number of compounds. Identification by partial least squares discriminant analysis (PLS-DA) combined with relative odor activity value (rOAV) screening yielded 22 candidate differential volatile organic compounds. Floral monoterpenes, including linalool, linalool oxide II and geraniol, were consistently higher in high-altitude teas, whereas most other volatiles varied primarily with cultivar rather than altitude. These chemical patterns are consistent with the sensory finding of stronger floral intensity in high-altitude samples. This study provides theoretical insights for cultivar selection and quality improvement of oolong tea grown in high-altitude regions.

## 1. Introduction

Tea [*Camellia sinensis* (L.) O. Kuntze], renowned for its unique flavor and health benefits, is one of the most popular non-alcoholic beverages worldwide [1,2]. Its rich flavor and many health-promoting effects have been linked to a diverse suite of bioactive constituents, including polyphenols such as catechins and flavonols, the purine alkaloid caffeine, the nonprotein amino acid, aroma volatiles and soluble sugars [3]. Overall, these constituents collectively underpin oolong tea’s sensory quality and are associated with antioxidant, neuroprotective, anxiolytic, and antibacterial activities [4,5,6]. In addition, its quality depends on multiple factors, including growing environment, cultivar, and processing techniques [7,8]. Among these, altitude is an important determinant of the composition and levels of secondary metabolites in fresh tea leaves, mainly through altitudinal gradients in environmental factors such as temperature, humidity, light and soil conditions [9]. Previous studies indicate that elevations of 800 m to 1200 m favor high-quality tea and are characterized, relative to lower altitudes, by lower temperatures, higher light intensity, and shorter sunshine duration [10]. Meanwhile, with increasing elevation, precipitation and incident solar and ultraviolet radiation generally increase [11]. In parallel, the soil environment also changes, with bacterial and fungal diversity and abundance often increasing with elevation and with marked shifts in community composition between low- and high-altitude sites [12]. These factors profoundly influence secondary metabolic pathways in tea plants, leading to alterations in the composition and concentration of secondary metabolites, which in turn modulate tea quality across diverse geographic regions [13,14]. Mounting evidence highlights the significant role of altitude in shaping tea flavor, offering valuable guidance for both scientific investigation and high-quality tea production.

In high-altitude environments, catechin and anthocyanin levels diminish, softening bitterness, while amino acids, such as L-theanine and glutamic acid, volatile terpenoids, like linalool, and fatty acids surge, imparting umami and floral aromas [10,15,16]. Moreover, high-altitude teas not only exhibit altered phenolic profiles but also demonstrate enhanced antioxidant activity and health benefits [17]. Previous studies have demonstrated that while the types of volatile compounds in tea are largely consistent across altitudes, differences in their concentrations and proportions play a crucial role in shaping the overall aroma profiles [18]. These subtle differences in metabolite composition may also influence tea brewing properties, further contributing to the perceived quality of high-mountain teas. Collectively, these observations shed light on the multifaceted influence of altitude on tea quality, demonstrating how changes in microclimatic conditions drive modifications in secondary metabolites, which in turn impact flavor. Yet, systematic research on oolong tea cultivars remains scarce, underscoring the need for deeper investigation into their responses to altitude variations.

Oolong tea, a traditional Chinese tea from Fujian Province, is prized for its distinctive flavor. The diverse topography and altitude gradients of the region create ecological heterogeneity, which in turn drives systematic variations in the volatile profiles of tea and ultimately influences its quality [19]. Moreover, evidence from prior research shows that oolong teas from different regions differ significantly in theaflavins, flavonoids, flavone glycosides, alkaloids, and pyrrolidones [20]. In addition, metabolomics has resolved differences across oolong tea grades, revealing quality-associated metabolites [21]. Common oolong tea cultivars, such as ‘Rougui’, ‘Shuixian’, ‘Qilan’, ‘Huangguanyin’, ‘Qidan’, and ‘Ruixiang’, are valued for their unique sensory profiles. For example, Rougui is celebrated for its intense floral-fruity aroma and sweet aftertaste [22]. Shuixian is noted for its robust body and lingering sweetness [23]. Given the intrinsic differences among these cultivars [24,25], substantial variation in their flavor profiles across altitudes is expected. Hence, a comprehensive analysis is essential to investigate the performance of different cultivars under high-altitude conditions.

This study aims to evaluate the flavor characteristics of six oolong tea cultivars across different elevations and to examine altitudinal differences in flavor-related composition and key volatile organic compounds (VOCs). The overall goal is to elucidate the compositional basis underlying flavor-quality differences between high- and low-altitude teas and to provide insights for high-mountain oolong tea production.

## 2. Results and Discussion

### 2.1. Characteristic Flavors of Oolong Teas from Different Altitudinal Regions

**Figure 1:**
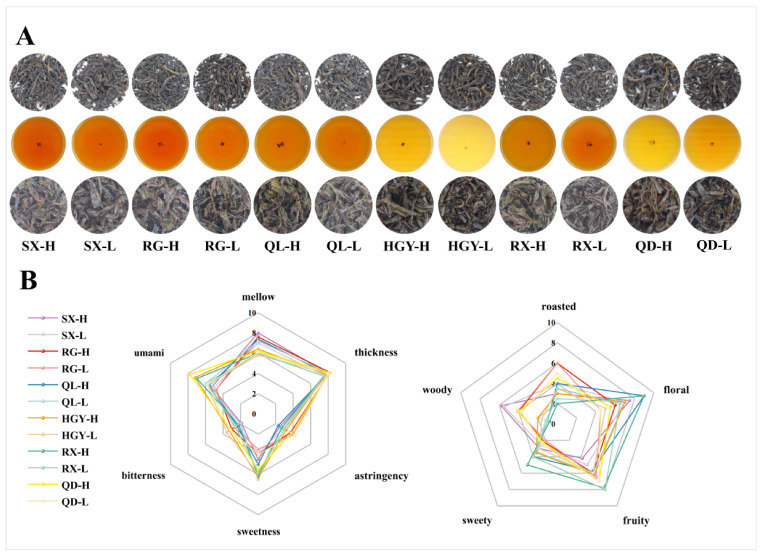
Sensory evaluation of six oolong tea cultivars at different altitudes: photographs of tea appearances, infusions, and infused leaves (**A**); quantitative descriptive analysis of taste and aroma characteristics (**B**).

The appearance and infusion of the six oolong tea cultivars are presented in Figure 1A. As shown in the figure, it is difficult to distinguish between high- and low-altitude tea samples based solely on their external morphology or the infusion. To gain a deeper understanding of their sensory characteristics, we conducted a comprehensive sensory evaluation focusing on taste and aroma attributes. Taste and aroma are the two most critical indicators of oolong tea quality, accounting for 35% and 30% of the overall score, respectively (GB/T 23776-2018) [26 ]. This assessment aimed to explore the potential influence of altitude on the organoleptic qualities of different tea cultivars. Quantitative descriptive analysis (Figure 1B) demonstrates that the sensory divergence among the tea samples is concentrated in three taste variables--mellowness, umami, and sweetness. The mellow scores of low-altitude tea samples ranged from 5.8 to 7.3, whereas cultivation in high-mountain gardens increased the scores to 6.0~8.0, leading to a smoother and more persistent taste. In terms of sweetness, the scores of low-altitude tea samples exhibited a broader range (3.5~6.5) with greater inter-varietal variation, whereas those of high-altitude teas were more concentrated (4.0~6.3), showing a distinct overall advantage in sweetness. Umami exhibits the greatest altitude-driven gain, expanding from 4.8~6.0 to 4.8~7.8 and underscoring the pronounced contribution of high-altitude conditions to savoury depth. By contrast, thickness exhibited the least variation, with scores ranging from 7.5 to 8.2, showing a maximum difference of only 0.7 points. Bitterness and astringency remain low overall. Nevertheless, cultivar-specific expression is evident, with RG, QD, and HGY registering the highest bitterness (2.7~3.0, 3.1~3.2, and 3.3~3.6, respectively) and corresponding astringency (3.7~4.0, 3.8~4.2, and 3.0~3.2), reaffirming their inherently more robust taste profiles.

The aroma dataset reveals pronounced cultivar effects superimposed on altitude-related modulation across five olfactory dimension--roasted, floral, fruity, sweet, and woody. RG and QD, irrespective of altitude, maintain consistently high roasted scores (4.5~6.0) accompanied by elevated fruity notes (6.0~7.0) but only moderate floral intensity (4.5~6.0), yielding a bouquet dominated by roasted and fruity notes. SX is characterised by an eminent woody note, peaking at 5.8 in the SX-H and delineating a mellow, wood-toned aromatic style. In contrast, QL-H and RX-H exhibited a pronounced altitude-dependent increase in floral intensity, each reaching the maximum score of 9.0, which represents an increase of approximately 2.0 and 2.5 points compared with their low-altitude counterparts, respectively. The sweetness attribute also increased, with QL-H and RX-H improving by 0.6 and 1.0 points, respectively, resulting in a clearly perceptible sweet floral aroma profile in the high-altitude tea samples for both cultivars. RX-H further combines the dataset’s highest fruity (7.8) and sweet (5.0) scores, resulting in the most delicate overall flavour architecture. HGY occupies an intermediate position, with balanced floral, fruity, and sweet attributes that confer a harmonised aromatic signature. In terms of altitude effects, high-elevation conditions broadly enhance floral and sweet aromas, but the intensity of each aroma attribute is ultimately determined by cultivar-specific characteristics.

Some studies have reported that higher altitude is associated with improvements in tea quality. For instance, high-altitude black teas were found to present intense honey-like notes that distinguish them from low-altitude teas [18]. Similarly, studies on sun-dried green tea in Yunnan demonstrated that teas cultivated at higher elevations were more likely to accumulate sweet mellow, and floral compounds [27]. With respect to oolong tea, altitude has been shown to enrich floral and honey-related precursors, thereby enhancing floral intensity [28]. Moreover, a metabolomics study confirmed that high-altitude teas generally exhibit reduced bitterness and astringency while showing elevated sweetness and umami [15]. However, our results also show that these improvements are not universal across tea cultivars. Responses to high elevation across sensory dimensions vary markedly among cultivars, with differences in both magnitude and direction. Consequently, altitude oriented cultivation and processing should be implemented at the cultivar level with targeted selection, multi site trials and process optimization, and the expected benefits should be confirmed by both sensory and chemical validation.

### 2.2. Significant Differences in Macro-Composition and Metabolites Between High- and Low-Altitude Oolong Teas

**Figure 2:**
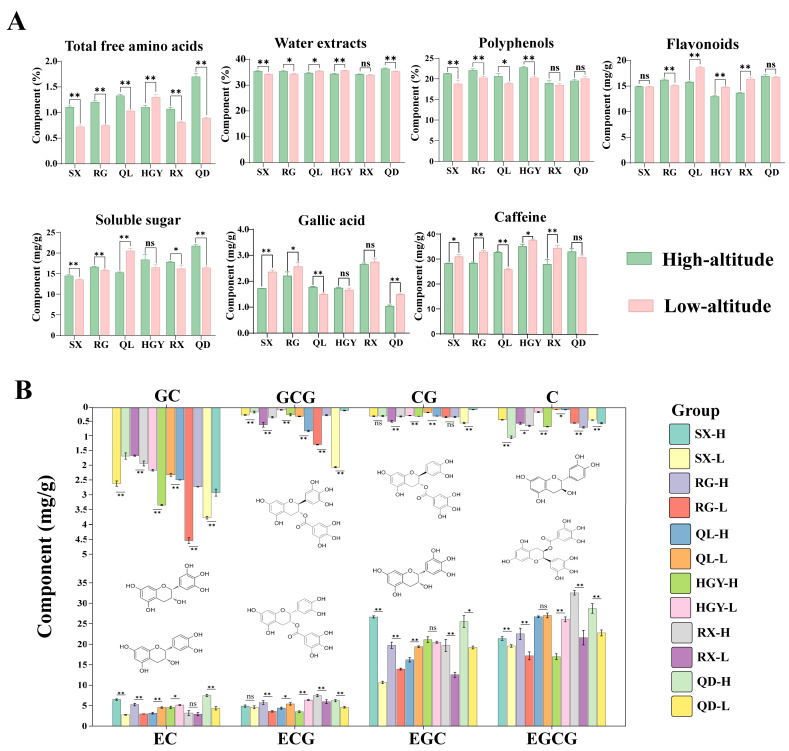
Effects of altitude on major quality-related compounds and catechin profiles in six oolong tea cultivars: comparison of macro-compositions (total free amino acids, water extracts, polyphenols, flavonoids, soluble sugar, gallic acid and caffeine) (**A**); comparison of catechin compositions (GC, GCG, CG, C, EC, ECG, EGC, EGCG) (**B**). Data are presented as mean ± standard deviation (*n* = 3). Significant differences between groups were determined by independent-samples *t*-test. * indicates *p* < 0.05, ** indicates *p* <0.01, "ns" indicates no significant difference.

As shown in Figure 2A, high altitude exerts multidimensional regulatory effects on the accumulation of key taste compounds in the six oolong tea cultivars (Table S1). Specifically, the content of free amino acids increased significantly with altitude (*p* < 0.01) in five cultivars (SX, RG, QL, RX, QD). Previous studies have shown that in green tea, the content of free amino acids increases significantly with elevation [29]. In oolong tea, high-altitude samples have also been found to contain higher levels of free amino acids [30]. In addition, studies have found that high-altitude environments, particularly their temperature and light conditions, can increase free amino acid content [10]. This suggests that high-altitude environments generally promote the accumulation of amino acids in tea leaves, providing a biochemical basis for the fresh taste of the tea. However, HGY exhibited lower free amino acid content at high altitude compared with low altitude (*p* < 0.01), likely reflecting cultivar-specific differences in amino acid accumulation under identical environmental stresses, as previously reported for tea [31]. As a comprehensive indicator of the total soluble substances in tea, the water extract mainly reflects the leaching amounts of polyphenols, amino acids, sugars, and a small amount of alkaloids, directly determining the concentration and fullness of the tea infusion [32]. In this study, the water extract content in high-altitude samples of SX, RG, and QD was significantly higher than that of their low-altitude counterparts. In contrast, QL and HGY showed a decreasing trend, and RX showed no significant difference. Regarding tea polyphenols, SX, RG, QL, and HGY showed significantly higher contents at high altitude, whereas RX and QD exhibited no significant changes. This phenomenon is likely attributable to the combined influence of unique alpine ecological factors such as low temperature, strong ultraviolet radiation, and a high ratio of diffuse light. It can therefore be speculated that enhanced UV and higher diffuse light fraction at high altitude could promote polyphenol accumulation in certain cultivars [14]. Flavonoid content showed a more pronounced interaction between genotype and environment, with an increase at high altitude in RG (*p* < 0.01), a decrease in QL (*p* < 0.01), HGY (*p* < 0.01), and RX (*p* < 0.01), and no significant change in SX and QD. Soluble sugars serve as common osmotic regulators and energy reserves for plants under cold stress. At high altitude, soluble sugar content was significantly higher than at low altitude in SX, RG, RX and QD. In contrast, QL exhibited a highly significant decrease in soluble sugar content at high altitude compared with low altitude, while HGY showed no significant difference between the two altitudes. Previous studies have shown that high-altitude environments are generally conducive to sugar accumulation in tea leaves under cold conditions [33]. For example, comparative analysis of fresh leaves from Longjing43 and Qunti tea trees grown at different elevations revealed that higher-altitude samples contained significantly greater amounts of soluble sugars [8], consistent with the trends observed in most cultivars in this study. The contents of gallic acid and caffeine showed no consistent trend between high- and low-altitude samples, with the direction and magnitude of change varying across cultivars.

Ester catechins are the primary contributors to the bitterness and astringency of tea [34]. Non-ester catechin possess lower bitterness and even help to enhance sweetness and smoothness in tea infusions [35]. In this study, all eight measured catechins responded to altitude in a cultivar dependent manner, with the direction of change varying by compound (see Figure 2B and Table S2). Notably, C increased consistently at high altitude in all six cultivars, indicating a stable altitude response. The other catechins (CG, EC, ECG, EGC, EGCG, GC and GCG) showed clear cultivar specificity. Across many catechin components, QL and HGY showed the same altitude response pattern, while SX, RG and QD showed the same but opposite pattern, and RX exhibited compound specific responses. These divergent shifts indicate that high altitude does not produce a uniform change in catechin profiles across cultivars, so the net effects on bitterness and astringency will be cultivar dependent. Practical decisions on altitude based cultivation or processing should therefore be made for each cultivar and confirmed by sensory testing to determine whether the chemical changes translate into the desired cup quality.

### 2.3. Aroma Composition of Oolong Teas Elucidated Through Headspace Solid-Phase Microextraction-Gas Chromatography-Mass Spectrometry (HS-SPME-GC-MS)

**Figure 3:**
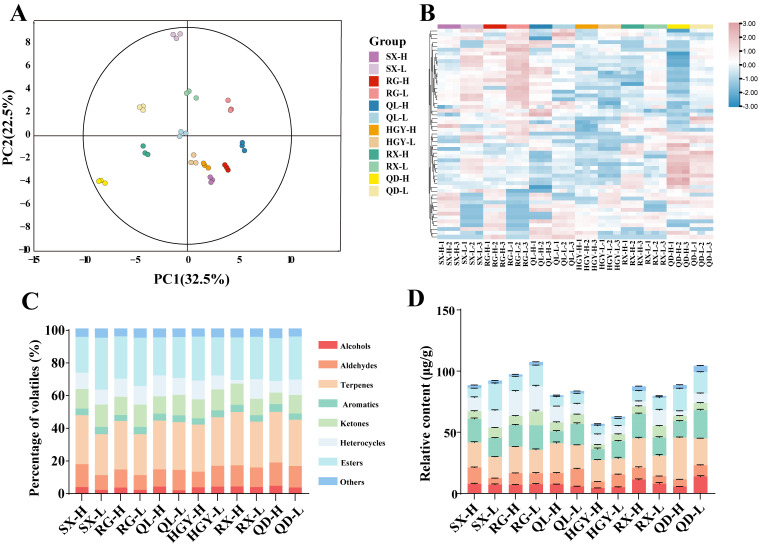
Detection and Analysis of Volatile Organic Compounds in Six Oolong Tea Cultivars at High and Low Altitudes: the PCA score plot of volatile organic compounds (**A**); cluster heatmap analysis of volatile organic compounds (**B**); proportion of volatile organic compound classification (**C**); relative content of volatile organic compounds (**D**).

To investigate the differences in aroma compounds of oolong tea grown at different altitudes, HS-SPME-GC-MS was employed for metabolomic analysis of 12 tea samples. A total of 55 common VOCs were identified (Table S3). Principal component analysis (PCA) and hierarchically clustered heatmaps were subsequently conducted to visualize the variation among samples. The PCA score plot (Figure 3A) revealed clear separation between high- and low-altitude samples, with the first two principal components (PC1 and PC2) explaining 32.5% and 22.5% of the total variance, respectively. This indicates a pronounced effect of altitude on the volatile compound profiles of oolong tea. Consistent grouping patterns were also observed in the heatmap clustering analysis (Figure 3B), further supporting the PCA results. Moreover, the close clustering of triplicate samples supports the stability and reliability of the HS-SPME-GC-MS method applied in this study. The identified volatile compounds were classified into eight categories: alcohols, aldehydes, esters, terpenes, aromatics, ketones, heterocyclic compounds, and others. As shown in Figure 3C, terpenes (25~33%) and esters (22~32%) consistently dominated the volatile profile across all samples, followed by aldehydes (9~14%), ketones (7~14%), and heterocyclic compounds (2~13%), while the remaining categories each accounted for less than 10%.

As shown in Figure 3D, it revealed that the volatile compound contents varied among these oolong teas, with RG and QD exhibiting relatively higher levels, while HGY showed the lowest relative volatile content (HGY-H: 56.91 μg/g, HGY-L: 62.78 μg/g). Notably, the relative volatile content of the high-altitude tea samples was lower than that of the low-altitude samples. Additionally, we investigated the content of volatiles across different categories. Terpenoids constitute the primary volatile compounds in oolong tea, typically contributing floral, fruity, and woody aromas. Among these, linalool, linalool oxide II, *α*-farnesene, (*E*)-nerolidol, and geraniol exhibited higher concentrations, collectively accounting for over 40% of the total terpenes in the tea samples, ranging from 47.62% to 83.93%. Additionally, (*+*)-limonene is a key aromatic component in tea. While it primarily imparts a fresh and crisp sensory profile in green tea, in oolong tea it often synergizes with other terpenes and esters to collectively create a refreshing floral-fruity aroma. Other compounds such as *α*-ionone, *β*-ionone, safranal, and (*E*)-*β*-farnesene also play important complementary roles in enhancing the layered complexity of oolong tea’s aroma. High levels of aromatic compounds were detected in all tea samples. Among these, indole is a common aromatic substance in tea leaves, making a key contribution to the floral and sweet characteristics of tea fragrance. At low concentrations, indole imparts a soft, jasmine-like fragrance to tea. Esters typically dominate the aromatic profile of oolong teas. Among these, methyl salicylate (wintergreen mint and sweet), hexyl hexanoate (sweet, fruity, and green), and (*Z*)-4-hexen-1-yl hexanoate (green and fruity) ranked as the top three esters in all samples. Aldehydes constitute an important class of volatile compounds in tea, significantly contributing to the overall aroma profile. Among core components, phenylacetaldehyde, benzaldehyde, and (*E*,*E*)-2,4-heptadienal are relatively abundant representatives within the aldehyde group, serving as key aldehyde constituents influencing aroma characteristics. Phenylacetaldehyde, generated from amino acid metabolism, possesses a low detection threshold and imparts a soft rose-like floral note. Phenylacetaldehyde is often accompanied by woody and nutty aromas. (*E*,*E*)-2,4-Heptadienal primarily originates from the oxidative degradation of fatty acids, contributing distinct grassy and fatty aromas. Across all samples, alcohol compounds ranged from 4.48 to 13.99 μg/g, predominantly exhibiting floral, sweet, and fruity characteristics. Phenethyl alcohol was the most abundant alcohol compound, significantly contributing to the overall aroma with its persistent rose-like fragrance. Compared to RX-L, RX-H showed a 3.48 μg/g increase in phenethyl alcohol content. However, QD exhibited the opposite trend, with QD-L containing 8.68 μg/g more phenethyl alcohol than QD-H. This discrepancy may relate to varying responses of aroma compounds to altitude conditions across different cultivars. Additionally, while ketones constituted a smaller proportion and concentration, most exhibited pleasant floral and sweet aromas. Methylheptenone, the most abundant ketone compound with fruity and citrus notes, exhibits higher levels in RX, likely contributing significantly to the variety’s aroma profile. Heterocyclic compounds act as crucial harmonizers within the aroma network, playing a pivotal role in enhancing aroma complexity and subtlety. Pyrrole compounds are volatile substances formed through the Maillard reaction between amino acids and reducing sugars during tea processing. These pyrrole compounds impart roasted, nutty, and caramel-like flavors, balancing the freshness of floral and fruity compounds to harmonize the overall aroma profile. The QD exhibited the highest total content of terpenes and esters at both high and low altitudes (52.59 μg/g and 39.68 μg/g, respectively), demonstrating a remarkable capacity for the accumulation of terpenes and esters. The SX showed a pronounced altitude response in ester accumulation, with a significantly higher ester content at low altitude (21.85 μg/g) compared to high altitude (7.74 μg/g), indicating that warm lowland environments may be more favorable for ester expression in this cultivar. Overall, as key contributors to oolong tea aroma quality, terpenes exhibited a general increasing trend in high-altitude samples. This trend was especially evident in the RX and QD cultivars, with increases of 7.42 μg/g and 12.28 μg/g, respectively, further highlighting the positive impact of high-altitude environments on aroma enhancement in certain cultivars. This phenomenon may be attributed to the marked upregulation of CsTPS and other terpene synthase genes in tea plants under high-altitude conditions, which in turn enhances the biosynthesis of terpene compounds [28,30]. In summary, both cultivar and altitude significantly shaped the composition and abundance of aroma compounds in oolong tea. High-altitude conditions favored terpene accumulation, while the variation of other aroma categories was largely cultivar-dependent, exhibiting diverse and dynamic characteristics.

### 2.4. Discovery of Key Aroma Markers Across Altitudes

**Figure 4:**
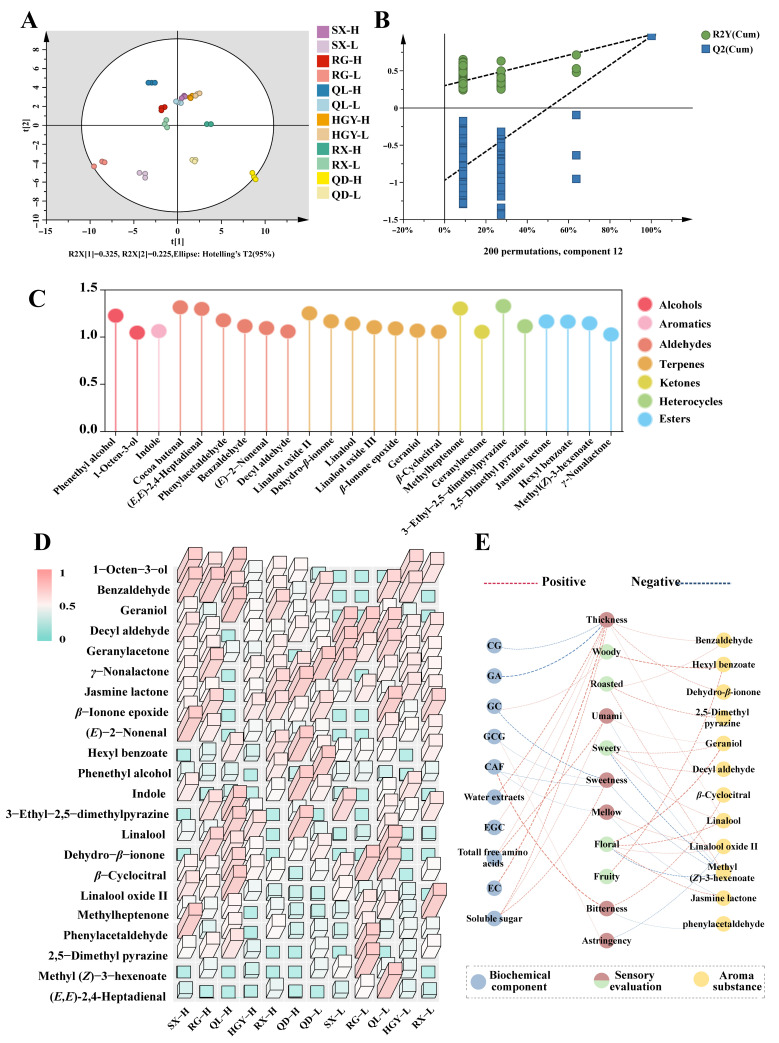
Multivariate Statistical Analysis and Key Volatile Organic Compounds of Six Oolong Tea Cultivars Grown at High and Low Altitudes: PLS-DA score plot of volatile compounds in 12 tea samples (**A**); permutation test for validating the PLS-DA model (**B**); volatile organic compounds with VIP values greater than 1 (**C**); heatmap of differential volatile organic compounds (VIP > 1 and rOAV > 1) between high- and low- altitude teas (**D**); correlation network illustrating the relationships among biochemical components, aroma substances, and sensory attributes (**E**).

To further explore VOCs associated with tea leaves produced at high and low altitudes, we employed a supervised partial least squares discriminant analysis (PLS-DA) model (Figure 4A). The model demonstrated a high predictive ability with a Q^2^ value exceeding 0.95, indicating that the volatile organic compound profiles were sufficient to simultaneously distinguish between tea cultivars and altitude levels. The cross-validation plot (Figure 4B) showed a good discriminatory performance, with an R^2^ intercept of 0.288 and a Q^2^ intercept of −1, confirming the robustness of the model and the absence of overfitting. These results suggest that tea leaves grown at different altitudes possess significantly distinct volatile chemical profiles, providing a solid foundation for the subsequent screening and interpretation of differential metabolites. In this study, we integrated multivariate statistical analysis with relative odor activity value (rOAV) calculations to identify potentially aroma-contributing compounds. In the PLS-DA model, the variable importance in projection (VIP) value was used to evaluate each variable’s contribution to classification [36]. A VIP > 1 indicates that the variable contributes more than average to the model and is considered an important discriminating feature (Figure 4C). To validate the variables selected by PLS-DA, an unsupervised PCA was performed using only the 24 VOCs with VIP > 1 [37]. The corresponding score and loading plots are provided in Supplementary Figure S1. The resulting score plot showed a separation pattern consistent with the PLS-DA, and the variables with the highest VIP scores also exhibited the largest absolute loadings on PC1 and PC2, confirming that the VIP-selected compounds are indeed the main contributors to sample discrimination. Simultaneously, rOAV was used as a key criterion for evaluating the aroma contribution of each compound, with rOAV > 1 suggesting a notable contribution to the overall aroma (Table S4) [38]. In this study, we found that the rOAV of linalool was the highest among all samples, indicating its significant role in the aroma of tea. Linalool is characterized by a sweet floral scent and is widely regarded as a key component contributing to the floral aroma profile of oolong tea [39]. The accumulation of linalool varied among different tea varieties, which is closely related to the inherent characteristics of the varieties. Zeng et al. [40] reported that the size of tea leaves is positively correlated with the linalool content, with larger-leaved varieties generally containing higher levels of linalool. Furthermore, the content of linalool was significantly affected by the processing of tea leaves. Studies have shown that under moderate light intensity, the spreading of tea leaves in green tea can significantly increase linalool content [41]. In the processing of yellow tea, it was also found that under conditions of 40 °C and 90% humidity, the linalool content was significantly higher than in naturally withered samples [42]. Mechanical damage also affects the linalool content in oolong tea. The process of shaking, such as in the -production of oolong tea, can induce the accumulation of linalool [43]. This suggests that shaking is a critical and fundamental processing step that affects the formation of quality in oolong tea. Similar to other types of oolong tea, linalool, (*E*)-2-nonenal, phenylacetaldehyde, 1-octen-3-ol, *β*-cyclocitral, jasmine lactone, geraniol, and (*E*,*E*)-2,4-heptadienal are key contributors to the aroma of oolong tea.

Based on these criteria, we identified 22 volatile compounds with VIP > 1 and rOAV > 1 as potential differential metabolites between high- and low-altitude tea samples (Table 1). These 22 compounds span multiple structural classes, including 2 alcohols, 5 aldehydes, 6 terpenoids, 2 ketones, 2 heterocyclic compounds, 4 esters and 1 aromatic compound. This diverse chemical profile indicates that the altitude-induced aroma differences are not attributable to a single class of compounds but rather to a combination of multiple volatile categories. Following the identification of these key volatiles, we visualized their abundance across samples using a heatmap (Figure 4D). Overall, high-altitude environments significantly promoted the accumulation of floral aroma compounds. Compared with low-altitude samples, many typical floral volatiles were consistently elevated in high-altitude samples across several cultivars. In line with this trend, previous studies also reported that aromatic volatiles such as benzy alcohol, phenylethanol, and acetophenone were more abundant in tea leaves from high-elevation regions, whereas aliphatic volatiles including 1-hexanol, 1-nonanol, and nonanoic acid were more prevalent in low-elevation tea orchards [44]. Furthermore, linalool and geraniol have been identified as key markers distinguishing teas from different altitudes [28], and in a comparative study of oolong teas produced in Tibet and Guangdong, PCA indicated that hotrienol and benzyl alcohol were the characteristic volatiles responsible for separating high- and low-altitude samples [30]. Taken together, these findings suggest a general pattern in which high-altitude teas tend to be enriched in many aromatic- and floral-related volatiles, whereas “green-note” volatiles are more characteristic of low-altitude teas Notably, linalool, linalool oxide Ⅱ and geraniol exhibited significantly increased levels (*p* < 0.05). These compounds typically carry sweet and floral notes and are recognized as key contributors to the floral aroma characteristic of oolong and other aromatic teas [45,46]. For instance, geraniol was found at significantly higher concentrations in all high-altitude tea samples compared to their low-altitude counterparts, suggesting enhanced biosynthesis or accumulation under high-altitude conditions. The accumulation of geraniol in high-altitude tea may be attributed to lower temperatures and stronger UV radiation in such environments. Previous studies have shown that lower temperatures can induce the release of aromatic terpenes such as linalool and geraniol [47]. Another study also demonstrated that short-term low-temperature treatment activates LOX genes, thereby promoting the biosynthesis of aroma compounds [48]. Additionally, enhanced UV radiation at high altitudes may lead to oxidative stress in plants, which in turn can upregulate the expression of terpene synthase genes, thereby promoting the biosynthesis of monoterpenes [49,50]. These mechanisms align with our findings. Additionally, appropriate processing methods, such as sun withering, shaking, and fixing, can enhance the accumulation of floral aroma compounds, including linalool, indole, and jasmine lactone [51]. This suggests that suitable processing techniques can also improve their aromatic quality.

Aside from the above compounds that showed clear and consistent altitude-related trends, the majority of other volatile metabolites exhibited no uniform change across high- and low-altitude samples among different cultivars. These variable patterns appear to be cultivar-dependent and are likely influenced by genetic background differences among tea varieties. To substantiate this cultivar effect beyond our dataset, prior studies have shown that even when processed into the same tea type, different cultivars often yield markedly distinct volatile profiles. The genetic background of tea plants can directly influence the accumulation of aroma compounds by regulating the expression of biosynthetic genes. For example, analysis of Congou black tea produced from seven different cultivars revealed significant differences in both the types and relative contents of volatile compounds [52]. Consistently, the Longjing population cultivar was relatively rich in esters, Longjing 43 contained higher levels of aldehydes, the Yingshuang cultivar showed higher proportions of heterocyclic volatiles, Longjing Changye accumulated more acids, while Jiukeng population tea was characterized by a higher abundance of alcohols [53]. Importantly, not all aroma differences are determined by cultivar. To clarify sources of variation, we distinguish between “varietal aromas,” which are primarily genotype-governed and relatively stable across standard processing, and “technological aromas,” which are predominantly generated or greatly amplified during processing. In oolong tea, (*E*)-nerolidol and *α*-farnesene are regarded as predominantly processing-derived and thus only weakly associated with cultivar traits, whereas (*Z*)-jasmone and *β*-caryophyllene are more closely tied to cultivar characteristics [54]. In addition, some changes in aroma compounds arise from genotype–environment interactions, and different cultivars do not respond to the environment in the same way. For example, Zhang et al. compared Shuixian and Rougui Wuyi rock teas from four subregions—Zhengyan (core area), Banyan, Waishan, and a high-elevation zone—and found that samples from Zhengyan received the highest sensory scores, while benzyl alcohol and hotrienol were identified as key volatiles distinguishing the four subregions [23]. Regarding cultivar-site suitability, multi-location trials in the Up-country tea region of Sri Lanka identified one genotype with the best adaptability and stability, whereas the other genotypes performed poorly in that region [55]. Along an altitude gradient, a recent study showed that only 16 metabolites changed in the same way across two cultivars, with most changes being cultivar-specific [15]. Therefore, in the comparison between high and low altitudes, altitude consistently elevates a small set of floral monoterpenes (linalool, geraniol and linalool oxides), whereas changes in most other volatiles are governed primarily by cultivar. In practice, this means that to achieve better aroma at high altitude, priority should be given to cultivars that are responsive to high-altitude conditions.

### 2.5. Correlation Analysis of Volatile and Nonvolatile Compounds with the Sensory Quality of Oolong Tea

Based on the correlation analysis in Figure 4E, the relationships between non-volatile compounds and VOCs and the sensory quality of tea were further investigated. The results show that thickness has a significant positive correlation with multiple biochemical components, particularly with EC, water extract, and soluble sugars, indicating that this sensory attribute is determined by the combined effect of several chemical components. In contrast, bitterness is significantly positively correlated with the content of caffeine and catechin components, suggesting that these compounds directly influence the astringency of the tea. Overall, volatile compounds, particularly linalool, geraniol, and jasmine lactone, are closely related to the floral aroma of tea. These volatile compounds, together with non-volatile substances, work synergistically to shape the complex aroma profile of the tea. These findings further highlight the complex interaction between biochemical components and sensory experiences, demonstrating that the formation of taste is not solely the result of individual compounds but rather the collaborative effect of multiple chemical substances.

## 3. Materials and Methods

### 3.1. Chemicals and Tea Samples

The following reagents are of analytical grade: Folin-Ciocalteu reagent, dipotassium hydrogen phosphate, disodium hydrogen phosphate, Ninhydrin, stannous chloride, sulfuric acid, sodium bicarbonate, ethanol, methanol and aluminum chloride. The Plant Soluble Sugar Content Assay Kit was purchased from Solarbio Technology Co., Ltd. (Beijing, China). L-Ascorbic acid, acetonitrile, and glacial acetic acid are of chromatographic grade and were purchased from Macklin Biochemical Technology Co., Ltd. (Shanghai, China). The standard compounds (+)-catechin (C), (−)-catechin gallate (CG), (−)-gallocatechin gallate (GCG), (−)-gallocatechin (GC), (−)-epicatechin (EC), (−)-epicatechin gallate (ECG), (−)-epigallocatechin (EGC), (−)-epigallocatechin gallate (EGCG), gallic acid and caffeine were sourced from Solarbio Technology Co., Ltd. (Beijing, China), with a purity of 98% or higher. Ethyl decanoate (chromatographic grade, Aladdin Biochemical Technology Co., Ltd., Shanghai, China) was used as the internal standard for GC-MS analysis of VOCs in oolong tea.

The study was conducted in Minqing County, Fujian, China at 26.47° N, 118.96° E. All sampling sites were at the same latitude. Therefore, latitude was not included as an independent variable in the analysis. Sampling covered two elevation bands, namely a high-altitude zone above 800 m and a low-altitude zone around 200 m. The tea gardens were established on granite-derived acidic red soils and were mostly on southeast-facing slopes. All sites were rain-fed, and supplemental irrigation was used only when warranted by weather conditions. The six oolong tea plant cultivars (*Camellia sinensis* var. *sinensis* ‘Rougui’, ‘Shuixian’, ‘Qilan’, ‘Ruixiang’, ‘Huangguanyin’, and ‘Qidan’) are cultivated at both elevation bands in Minqing County. The identification numbers of the tea plant varieties are detailed in Table S5. Fresh tea samples were collected from Fujian Qilinshan Tea Industry Development Co., Ltd (Fuzhou, Fujian, China). In the spring of 2025, the fresh leaves were harvested to the same standard (one bud with three to four leaves), with diseased or insect-damaged leaves removed. Fresh leaves from each lot were processed by master tea makers following conventional procedures, which included withering, shaking, fixing, kneading, drying and baking. Briefly, solar-withering was carried out for 40 min at 22~25 °C and 50~55% relative humidity. After withering, the leaves were transferred to a 6CZQ-110 green-making machine. Six shaking cycles were performed in sequence: 1 min at 8 rpm, 2 min at 8 rpm, 5 min at 8 rpm, 3 min at 12 rpm, 8 min at 12 rpm, and 13 min at 16 rpm. After each shaking, the leaves were rested for 1 h and then air-blown. The leaves were fixed at 260~280 °C for 7 min, cooled to 40~50 °C, and rolled for 6 min. Two-stage drying followed: 95 °C for 30 min and then 100 °C for 30 min. Finally, the dried tea was charcoal-roasted on bamboo sieves at 110 °C, turned once every hour, for a total of 13 h. To ensure clarity, each sample was labeled using a combination of cultivar abbreviation (SX for Shuixian, RG for Rougui, QL for Qilan, RX for Ruixiang, HGY for Huangguanyin, and QD for Qidan) and altitude designation (‘H’ for high altitude, ‘L’ for low altitude). The tea samples were divided into two parts. One part was used for sensory evaluation, while the other was ground into powder for composition analysis.

### 3.2. Sensory Evaluation

The panel consisted of seven members (three males and four females) aged 23–51 years, with over five years of experience in tea evaluation. All members underwent systematic training and provided informed consent prior to participating in the experimental procedures. The tea samples were evaluated by a trained panel following the tea sensory evaluation standard (GB/T 23776-2018) [26]. Specifically, 5.00 g of each tea sample was weighed and placed in a 110 mL tea evaluation cup, then quickly filled with boiling water. The tea infusion was obtained at 2, 3 and 5 min, respectively. The expert panel unanimously agreed that the taste attributes of the tea samples were characterized as mellow, umami, bitter, sweet, astringent, and thick, while the aroma attributes were roasted, floral, fruity, sweet, and woody. The intensities of both taste and aroma attributes were rated on a 0 to 10 scale, with 0 indicating none or imperceptible presence and 10 representing an extremely strong level.

### 3.3. Determination of Major Non-Volatile Components

The water extract content of the tea samples was determined according to the Chinese National Standard (GB/T 8305-2013) [56]. The total flavonoid content was measured using the aluminum chloride colorimetric method. The total polyphenols content was quantified by oxidation with the Folin-Ciocalteu reagent, following the Chinese National Standard (GB/T 8313-2018) [57]. The soluble sugar content was analyzed using the anthrone colorimetric according to the instructions of the Plant Soluble Sugar Content Assay Kit (Solarbio, Beijing, China). Free amino acids were measured by spectrophotometry by the Chinese National Standard (GB/T 8314-2013) [58].

### 3.4. High-Performance Liquid Chromatography (HPLC) Analysis

Catechins, caffeine and gallic acid were determined using a HPLC system (Alliance E2695 Separations Module coupled with a 2998 photodiode array Detector (PAD), Waters Corp., 34 Maple Street, Milford, MA, USA) equipped with an Agilent Eclipse XDB-Phenyl column (5 μm, 4.6 × 250 mm, Agilent Technologies, Santa Clara, CA, USA). We followed the method of Tukhvastshin et al. [59] with minor modifications, as detailed below. Specifically, 0.2 g of tea powder was extracted with 70% methanol in a 70 °C water bath for 10 min. After centrifugation (10 min, 3500 r/min), the supernatant was collected and the extraction was repeated once. The combined supernatant was diluted fivefold with a stabilizing solution (50 mL acetonitrile and 0.2 g ascorbic acid in purified water, made up to 500 mL), filtered through a 0.45 μm membrane, and analyzed by HPLC. Each tea sample was measured in triplicate. The separation of chemical compounds was performed using gradient elution with solvent A (9.0% acetonitrile and 2.0% glacial acetic acid, *v*/*v*) and solvent B (80% acetonitrile, *v*/*v*) as the mobile phase. The flow rate was set to 1 mL/min. The injection volume was 10 μL. The gradient program started with 100% solvent A from 0 to 11.5 min, followed by a linear transition from 11.5 to 15.5 min, where solvent A decreased to 70% and solvent B increased to 30%. This composition was maintained until 26 min, then gradually returned to the initial condition with solvent A rising to 100% and solvent B decreasing to 0% from 26 to 30 min and held until the end of the run at 36 min. UV detection was performed at 278 nm using the PDA detector. The HPLC chromatograms are shown in Figure S2, and the retention times of each compound are listed in Table S6.

### 3.5. HS-SPME-GC-MS Detection of VOCs

For the analysis of VOCs, 0.5 g of tea powder was placed into a 20 mL headspace vial, followed by the addition of 4 mL NaCl saturated solution of cold distilled water and 10 μL of internal standard solution (ethyl decanoate, 10 μg/mL). The experiment was performed with three replicates per treatment (*n* = 3). At the time of solid-phase microextraction (SPME), the vials were incubated at 85 °C for 5 min to equilibrate, and then a 50/30 μm DVB/CAR/PDMS fiber (Supelco, Bellefonte, PA, USA) was exposed to the headspace at 85 °C for 45 min. VOCs were thermally desorbed at 250 °C for 5 min in splitless mode.

GC-MS analysis was performed using a Shimadzu GCMS-QP2020 NX system (Shimadzu, Kyoto, Japan) equipped with an SH-PolarWax capillary column (30 m × 0.25 mm × 0.25 μm, Shimadzu). The oven temperature was programmed as follows: initial temperature at 40 °C (held for 3.50 min), ramped at 5 °C/min to 100 °C (held for 2 min), then at 4 °C/min to 180 °C (held for 5 min), followed by 10 °C/min to 255 °C (held for 5 min), with a total run time of 57.50 min. Helium was the carrier gas at a constant flow rate of 1.2 mL/min. The injection port and ion source temperatures were set at 250 °C, and the transfer line temperature was maintained at 280 °C. Electron ionisation (EI) was applied at 70 eV, and mass spectra were acquired in scan mode. VOCs were identified by matching their mass spectra against the NIST library and further confirming with the linear retention index.

### 3.6. Relative Quantification of VOCs and Evaluation of rOAVs

The relative quantification of VOCs was conducted based on the ratio of their chromatographic peak areas to that of the internal standard. The rOAVs were determined by calculating the ratio of each VOC’s relative concentration in the tea samples to its corresponding odor threshold in water. This approach allows for the evaluation of each VOC’s relative contribution to the overall aroma profile of the tea samples. The formula is

$$rOAV=\frac{C}{OT}$$

where *C* represents the relative concentration of each compound (μg/g) and *OT* indicates its detection threshold in water (μg/g).

### 3.7. Statistical Analysis

The radar plot was generated using Origin software (version 2025, OriginLab Corporation, Northampton, MA, USA), and the bar chart was created using GraphPad Prism (version 10.1.2, GraphPad Software, San Diego, CA, USA). Differences in metabolites among tea samples were visualized as heatmaps using TBtools software (version 2.315). PCA and PLS-DA were conducted using SIMCA software (version 18.0, Umetrics, Umea, Sweden). Pearson correlations were computed in R 4.3 (R Foundation for Statistical Computing, Vienna, Austria) using the Hmisc package, retaining edges with an absolute correlation coefficient |r| > 0.7 and *p* < 0.05. The resulting co-occurrence (correlation) network was visualized in Cytoscape 3.9.1. Unless otherwise specified, all other figures were generated in R version 4.3. Statistical analyses were performed using *t*-test with SPSS software (version 27.0.1, SPSS Inc., Chicago, IL, USA). Differences were considered statistically significant when the *p*-value was less than 0.05.

## 4. Conclusions

Overall, across six oolong tea cultivars, sensory differences between altitudes were expressed mainly as increases in umami, sweetness, and floral intensity. High-altitude samples generally performed better in these attributes. At the chemical level, high altitude increased free amino acids, tea polyphenols and soluble sugars in most cultivars. Flavonoids, water extract and individual catechins showed cultivar-dependent responses. Notably, the non-ester catechin C increased consistently with altitude in all six cultivars. HS-SPME-GC-MS showed that the volatile classes were dominated by terpenes and esters, and linalool, (*E*)-2-nonenal, phenylacetaldehyde, 1-octen-3-ol, *β*-cyclocitral, jasmine lactone, geraniol and (*E*,*E*)-2,4-heptadienal were key contributors to the aroma of oolong tea. PLS-DA robustly separated samples by altitude and cultivar. Combined with rOAV, this approach identified 22 differential markers. Among these, floral monoterpenes such as linalool, linalool oxide II and geraniol accumulated at high altitude, matching the stronger floral perception in the sensory evaluation. Most other volatiles displayed clear cultivar specificity, which highlights the central role of genotype-by-environment interactions in shaping aroma profiles. From an applied perspective, altitude-oriented production should prioritise cultivars that respond favourably and stably to high altitude. These findings motivate targeted optimization of processing, with refined solar-withering, shaking and fixing, to amplify the advantageous flavor attributes of high-altitude teas.

## Figures and Tables

**Table 1:** 22 key volatile compounds with VIP > 1 and rOAV > 1 and their odor description and type.

Compounds	Odor Description	Odor Type	VIP Score ^a^
Phenethyl alcohol	Honey, sweet, rose, fresh	Floral	1.2272 ^a^
1-Octen-3-ol	Cucumber, earth, fatty, mushroom	chemical	1.0468 ^a^
Indole	Floral, animal-like	Floral	1.0638 ^a^
(E,E)-2,4-Heptadienal	Fatty, green, oily, cinnamon-like	Chemical	1.2995 ^a^
phenylacetaldehyde	Green, floral, sweet, cocoa	Green	1.1796 ^a^
Decyl aldehyde	Fatty, orange peel, tallow	Waxy	1.0611 ^a^
(E)-2-Nonenal	Floral, green, citrus, waxy	Floral	1.0964 ^a^
Benzaldehyde	Caramel, fruity, bitter almond, burnt sugar	Roasted	1.1166 ^a^
Linalool oxide II	Sweet, floral, creamy	Floral	1.2539 ^a^
Linalool	Floral, sweet	Floral	1.1423 ^a^
β-Cyclocitral	Tropical, saffron, herbal, tobacco-like	Woody	1.0565 ^a^
Geraniol	Rose-like, sweet, honey-like	Floral	1.0693 ^a^
β-Ionone epoxide	fruit, sweet, woody	Fruity	1.0931 ^a^
Dehydro-β-ionone	Violet, woody, raspberry	Floral	1.1675 ^a^
Methylheptenone	Pepper, mushroom, rubber	Citrus	1.3031 ^a^
Geranylacetone	Fresh, green, floral, rose	Floral	1.0552 ^a^
3-Ethyl-2,5-dimethylpyrazine	Cocoa-like, roasted, nutty	Roasted	1.3297 ^a^
2,5-Dimethyl pyrazine	Nutty, coffee, cocoa-like	Roasted	1.1156 ^a^
Jasmine lactone	Lactonic, creamy, sweet, floral	Floral	1.1671 ^a^
*γ*-Nonalactone	Sweet, creamy, coconut-like	Fruity	1.0293
Hexyl benzoate	Woody, green, piney	Woody	1.1647 ^a^
Methyl (*Z*)-3-hexenoate	Fruit-like, floral	Fruity	1.1466 ^a^

Odor description and odor type found in the literature with the database (https://www.flavornet.org/flavornet.html, accessed on 17 July 2025; https://www.thegoodscentscompany.com, accessed on 18 July 2025). ^a^ *p* < 0.05.

## Data Availability

The original contributions presented in this study are included in the article/Supplementary materials. Further inquiries can be directed to the corresponding author.

## Supplementary Materials

The following supporting information can be downloaded at: https://www.mdpi.com/article/10.3390/plants15010023/s1, Table S1: Information on total free amino acids, water extracts, polyphenols, flavonoids, soluble sugars, gallic acid and caffeine; Table S2: Information on catechin components; Table S3: Data for the 55 common VOCs; Table S4: Information on compounds with rOAV > 1; Table S5: plant identification number; Table S6: HPLC retention times; Figure S1: PCA score and loading plots based on VIP-selected variables (VIP > 1); Figure S2: The HPLC chromatograms.
