# Supplementary material for: Comparison of Flavor Characteristics and Metabolite Basis of Oolong Tea from Six Different Tea Plant Cultivars Under High- and Low-Altitude Conditions
Source: Plants (Basel). 2025 Dec 21;15(1):23. doi: 10.3390/plants15010023 (PMC12787703; doi:10.3390/plants15010023)
Supplement: Supplementary file 1 [file plants-15-00023-s001.zip › Supplementary Figures.pdf]

**This PDF file includes:** Supplementary Figures S1–S2

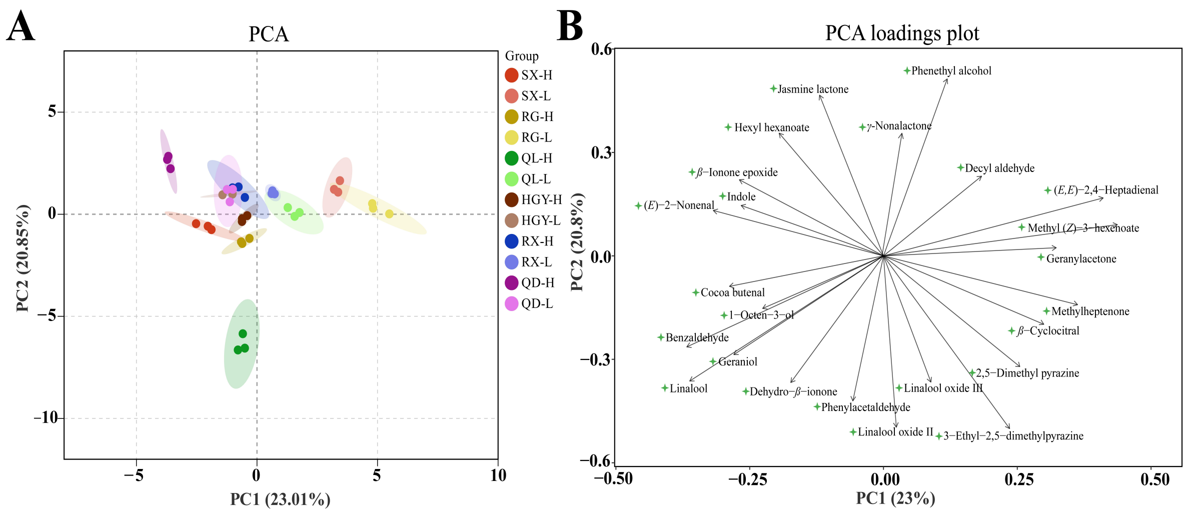


**Figure S1.** PCA score (A) and loading plots (B) based on VIP-selected variables (VIP > 1)

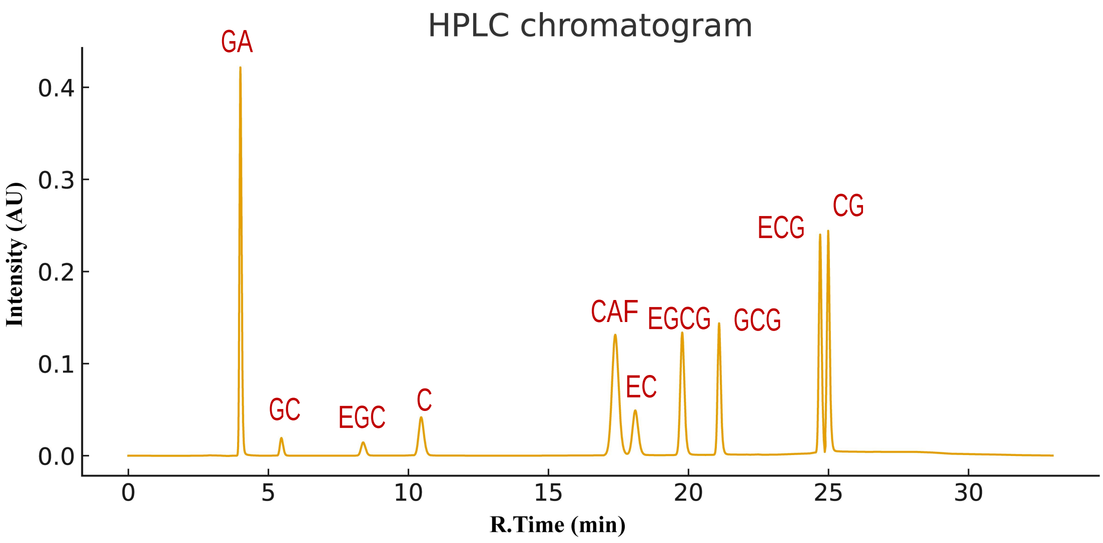


**Figure S2.** The HPLC chromatograms of the standards
